# Public Perspectives on COVID-19 Vaccine Prioritization

**DOI:** 10.1001/jamanetworkopen.2021.7943

**Published:** 2021-04-09

**Authors:** Govind Persad, Ezekiel J. Emanuel, Samantha Sangenito, Aaron Glickman, Steven Phillips, Emily A. Largent

**Affiliations:** 1 University of Denver Sturm College of Law, Denver, Colorado; 2 Department of Medical Ethics and Health Policy, University of Pennsylvania Perelman School of Medicine, Philadelphia; 3 Fels Institute of Government, University of Pennsylvania, Philadelphia; 4 Penn Program on Opinion Research and Election Studies, University of Pennsylvania, Philadelphia; 5 Leonard Davis Institute of Health Economics, Department of Medical Ethics and Health Policy, University of Pennsylvania Perelman School of Medicine, Philadelphia; 6 COVID Collaborative, Washington, DC

## Abstract

**Importance:**

As COVID-19 vaccine distribution continues, policy makers are struggling to decide which groups should be prioritized for vaccination.

**Objective:**

To assess US adults’ preferences regarding COVID-19 vaccine prioritization.

**Design, Setting, and Participants:**

This survey study involved 2 independent, online surveys of US adults aged 18 years and older, 1 conducted by Gallup from September 14 to 27, 2020, and the other conducted by the COVID Collaborative from September 19 to 25, 2020. Samples were weighted to reflect sociodemographic characteristics of the US population.

**Exposures:**

Respondents were asked to prioritize groups for COVID-19 vaccine and to rank their prioritization considerations.

**Main Outcomes and Measures:**

The study assessed prioritization preferences and agreement with the National Academies of Science, Engineering, and Medicine’s Preliminary Framework for Equitable Allocation of COVID-19 Vaccine.

**Results:**

A total of 4735 individuals participated, 2730 (1474 men [54.1%]; mean [SD] age, 59.2 [14.5] years) in the Gallup survey and 2005 (944 men [47.1%]; 203 participants [21.5%] aged 55-59 years) in the COVID Collaborative survey. In both the Gallup COVID-19 Panel and COVID Collaborative surveys, respondents listed health care workers (Gallup, 93.6% [95% CI, 91.2%-95.3%]; COVID Collaborative, 80.0% [95% CI, 78.0%-81.9%]) and adults of any age with serious comorbid conditions (Gallup, 78.6% [95% CI, 75.2%-81.7%]; COVID Collaborative, 72.9% [95% CI, 70.7%-74.9%]) among their 4 highest priority groups. Respondents of all political affiliations agreed with prioritizing Black, Hispanic, Native American, and other communities that have been disproportionately affected by COVID-19 (Gallup, 74.2% [95% CI, 70.6%-77.5%]; COVID Collaborative, 84.9% [95% CI, 83.1%-86.5%]), and COVID Collaborative respondents were willing to be preceded in line by teachers and childcare workers (92.5%; 95% CI, 91.2%-93.7%) and grocery workers (85.9%; 95% CI, 84.2%-87.5%). Older respondents in both surveys were significantly less likely than younger respondents to prioritize healthy adults aged 65 years and older among their 4 highest priority groups (Gallup, 23.7% vs 39.1% [χ^2^ = 2160.8; *P* < .001]; COVID Collaborative, 23.3% vs 28.8% [χ^2^ = 5.0198; *P* = .03]). COVID Collaborative respondents believed the 4 most important considerations for prioritization were preventing COVID-19 spread (78.4% [95% CI, 76.3%-80.3%]), preventing the most deaths (72.1% [95% CI, 69.9%-74.2%]), preventing long-term complications (68.9% [66.6%-71.9%]), and protecting frontline workers (63.8% [95% CI, 61.5%-66.1%]).

**Conclusions and Relevance:**

US adults broadly agreed with the National Academies of Science, Engineering, and Medicine’s prioritization framework. Respondents endorsed prioritizing racial/ethnic communities that are disproportionately affected by COVID-19, and older respondents were significantly less likely than younger respondents to endorse prioritizing healthy people older than 65 years. This provides reason for caution about COVID-19 vaccine distribution plans that prioritize healthy adults older than a cutoff age without including those younger than that age with preexisting conditions, that aim solely to prevent the most deaths, or that give no priority to frontline workers or disproportionately affected communities.

## Key Points

**Question:**

Which groups does the public believe should be prioritized for COVID-19 vaccine access?

**Findings:**

In this survey study of 4735 US adults, respondents of all demographic and political affiliations agreed with prioritizing health care workers, adults of any age with serious comorbid conditions, frontline workers (eg, teachers and grocery workers), and Black, Hispanic, Native American, and other communities that have been disproportionately affected by COVID-19. Older adult respondents were less likely than younger respondents to list healthy people older than 65 years as 1 of their top 4 priority groups.

**Meaning:**

These findings suggest that the US public agrees with the high-priority groups proposed by the National Academies of Science, Engineering, and Medicine but appears to disagree with approaches advanced by others that prioritize older adults but not essential workers or disproportionately affected communities.

## Teaser

This survey study assesses US adults’ preferences regarding which groups should receive COVID-19 vaccines first.

## Introduction

Since the US Food and Drug Administration’s issuance of emergency use authorizations for the Pfizer-BioNTech and Moderna COVID-19 vaccines, mass vaccination efforts have begun across the US.^1^ Because demand for COVID-19 vaccines exceeds the supply, phased distribution has been necessary, and prioritization, and targeted outreach will likely continue even once eligibility is open to all. The Centers for Disease Control and Prevention’s (CDC) Advisory Committee on Immunization Practices (ACIP) has—largely consistent with the distribution plan set forth in the National Academies for Science, Engineering, and Medicine’s (NASEM) Preliminary Framework for Equitable Allocation of COVID-19 Vaccine—recommended that health care personnel and long-term care facility residents receive the initial doses (phase 1a), followed next by people older than 75 years and frontline workers (phase 1b), and then by people aged 65 to 74 years, those with high-risk medical conditions, and other essential workers (phase 1c).^2,3,4,5^ Federal, state, local, and tribal jurisdictions are drawing on, but often varying from, these nonbinding CDC ACIP recommendations to plan and implement distribution.^6,7,8^ In fact, access to COVID-19 vaccination has proven highly dependent on where one lives, as distribution plans continue to evolve and to diverge from both CDC ACIP recommendations and from one another.^9^

With COVID-19 vaccination underway, policy makers are confronting numerous open questions about how to allocate vaccines. What should be the relative prioritization of essential workers, healthy older adults, and people with high-risk medical conditions?^4^ Should prisoners and prison guards be prioritized before grocery store workers?^10^ Should socioeconomically disadvantaged communities disproportionately affected by COVID-19 receive priority?^11^ What about COVID-19 vaccine trial participants who received a placebo?^12^ Although public opinion does not determine right and wrong, public engagement can aid in answering questions like these, help to identify gaps between public values and proposed prioritization schemes, and estimate the perceived legitimacy of allocation policies.^13,14^ Public views can be particularly helpful in prioritization between groups that are otherwise equally ranked on ethical grounds. Prior surveys^15,16,17^ have examined preferences regarding scarce resource allocation in the pandemic, but few have focused specifically on COVID-19 vaccine allocation. To address this gap, we surveyed 2 representative samples of US adults about COVID-19 vaccine allocation priorities.

Our study builds on prior surveys in key ways. First, we required respondents to rank priority groups comparatively, which more accurately reflects vaccine allocation under conditions of scarcity. Second, we disaggregated certain essential workers, such as teachers, restaurant workers, and grocery store workers. Third, we surveyed attitudes toward different allocation considerations or principles focused specifically on vaccines, rather than on scarce resources broadly.

## Methods

The University of Pennsylvania Institutional Review Board exempted this survey study. Participants consented through the survey websites. This study follows American Association for Public Opinion Research (AAPOR) reporting guideline.

### Gallup COVID-19 Panel

From September 14 to 27, 2020, Gallup conducted a nationwide online survey, in English, of US adults aged 18 years and over. The Gallup COVID-19 Panel is a probability-based panel of US adults selected using address-based sampling methods and random-digit-dial telephone interviews that cover landline and cellular telephones. Using Current Population Survey data, the study sample was weighted by age, gender, race/ethnicity, education, and US Census region, to be demographically representative of the US adult population. The survey achieved a response rate of 39% using the American Association for Public Opinion Research Response Rate Calculation (AAPOR RR 1). Gallup offered survey respondents $1 for completing the survey. The survey questions are shown in eAppendix 1 in the Supplement.

### COVID Collaborative

From September 19 to 25, 2020, Hart Research conducted a nationwide online survey, in English, among US adults aged 18 years and older on behalf of the COVID Collaborative, a national collaboration of experts in health, education, and economics focused on developing consensus recommendations related to the COVID-19 pandemic. Respondents were recruited from an online opt-in, nonprobability panel. Quotas were set and slight weights were applied to ensure that the sample was representative of adults overall and within subgroups by key demographic variables. The survey achieved a participation rate of 93% (defined as the number of completed surveys, terminates, or over quota, divided by the number of respondents who entered the survey) and a completion rate (completed surveys divided by number of respondents who entered the survey) of 42%. The AAPOR RR1 is not applicable, as the number of people presented with the survey in the opt-in panel is not known. Hart Research offered respondents an incentive that averaged $1.57 per completed survey. The survey questions are shown in eAppendix 2 in the Supplement. The surveys were developed in collaboration with Gallup and Hart Research staff, respectively. Gallup COVID-19 Panel questions were pretested in small panels before fielding. Survey respondents provided demographic data, with response options provided by Gallup and Hart Research. Questions about race/ethnicity were included because people of color have faced disproportionate COVID-19 burden due to exacerbation of longstanding health disparities and the effects of structural racism.

### Statistical Analysis

Respondents’ answers were compared using χ^2^ tests accounting for survey weights (Table 1). Statistical significance was set at α = .05 for 2-tailed tests. Analyses were conducted using R statistical software version 4.0.2 (R Project for Statistical Computing). The demographic characteristics of both samples were weighted by gender, race, education, and political affiliation to reflect the US population. Weighted distributions for the Gallup COVID-19 panel were 51.3% women; 73.2% White and 26.8% Black, Asian, Hispanic, and other ethnicities (including Native American, Alaska Native, Native Hawaiian, or Pacific Islander); and 41.3% Democratic, 29.6% Republican, 24.9% Independent, and 4.2% other political affiliation individuals. The Gallup panel was weighted to have 34.84% of individuals with at least a bachelor’s degree and 65.2% without. Weighted distributions for the COVID Collaborative survey were 51.9% women; 63.0% White and 37.0% Black, Asian, Hispanic, and other ethnicity individuals; and 43.0% Democratic, 35.0% Republican, and 22.0% Independent affiliation individuals. The COVID Collaborative survey was weighted to have 33.0% of individuals with at least a bachelor’s degree and 67.0% without (Table 1 and eTable 1 in the Supplement).

**Table 1.** Respondent Demographic Characteristics^a^

Characteristic	Respondents, No. (%)			
	Gallup COVID-19 panel		COVID Collaborative	
	Unweighted	Weighted	Unweighted	Weighted
Gender				
Female	1250 (45.9)	1350 (51.3)	1060 (52.9)	1041 (51.9)
Male	1474 (54.1)	1284 (48.7)	944 (47.1)	962 (48.0)
Race				
Asian	47 (1.7)	20 (0.7)	110 (5.5)	111 (5.5)
Black	118 (4.3)	288 (10.8)	277 (13.8)	261 (13.0)
Hispanic	148 (5.4)	403 (15.1)	308 (15.4)	320 (16.0)
White	2385 (87.7)	1951 (73.2)	1269 (63.3)	1263 (63.0)
Native American, Alaska Native, Native Hawaiian, or Pacific Islander	22 (0.8)	5 (0.2)	41 (2.0)	49 (2.4)
Political affiliation				
Democratic	1121 (41.4)	1087 (41.3)	849 (42.4)	861 (43.0)
Independent	678 (25.0)	654 (24.9)	458 (22.9)	441 (22.0)
Republican	829 (30.6)	778 (29.6)	698 (34.8)	702 (35.0)
Other party	80 (3.0)	111 (4.2)	NA	NA
Education				
Less than bachelor’s degree	1155 (42.4)	1718 (65.2)	1316 (65.7)	1343 (67.0)
Bachelor’s degree and above	1569 (57.6)	915 (34.8)	689 (34.4)	661 (33.0)

Abbreviation: NA, not applicable.

^a^ Some groups will not add up to the full sample total because of nonresponse or refusal.

## Results

### Highest Priority Groups

A total of 4735 individuals participated, 2730 (1474 men [54.1%]; mean [SD] age, 59.2 [14.5] years) in the Gallup online survey and 2005 (944 men [47.1%]; 203 participants [21.5%] aged 55-59 years) in the COVID Collaborative online survey. Both Gallup COVID-19 Panel and COVID Collaborative respondents prioritized health care workers and adults of any age with serious comorbid conditions (Table 2). In the Gallup COVID-19 panel, 93.6% of respondents (95% CI, 91.2%-95.3%) ranked health care workers and 78.6% (95% CI, 75.2%-81.7%) ranked adults of any age with serious health conditions among their top 4 priority groups. In the COVID Collaborative sample, 80.0% of respondents (95% CI, 78.0%-81.9%) ranked health care workers and 72.9% (95% CI, 70.7%-74.9%) ranked “people with serious medical conditions that make them more likely to have complications or die from COVID-19” among their top 4 priority groups.

**Table 2.** Highest Priority Groups for COVID-19 Vaccine Allocation

Group	Respondents, No. (%) [95% CI]		
	Gallup COVID-19 panel, top 4 priority group	COVID collaborative	
		Top 4 priority group	Willingness to wait behind
High-risk populations			
Nursing home residents and staff	NA	1114 (55.0) [52.6-57.3]	1909 (96.2) [95.1-97.0]
Individuals with underlying comorbidities	2059 (78.6) [75.2-81.7]	1449 (72.9) [70.7-74.9]	1780 (95.6) [94.4-96.5]
People of color and other communities with higher COVID-19 burden	NA	791 (39.5) [37.2-41.9]	1587 (84.9) [83.1-86.5]
Adults in group settings	1370 (45.7) [41.8-49.7]	NA	NA
Employment-based groups			
Health care workers	2526 (93.6) [91.2-95.3]	1615 (80.0) [78.0-81.9]	1914 (96.6) [95.6-97.4]
Teachers and childcare workers	1318 (48.3) [44.3-52.2]	653 (32.2) [30.0-34.4]	1805 (92.5) [91.2-93.7]
Nonhealth essential workers	1245 (47.1) [43.2-51.1]	NA	NA
Grocery store workers	NA	300 (14.4) [12.8-16.2]	1700 (85.9) [84.2-87.5]
Restaurant, bar, and gym workers	NA	181 (8.9) [7.7-10.4]	1456 (74.2) [72.1-76.2]
People in prisons and prison guards	NA	161 (8.2) [6.9-9.6]	1103 (56.4) [54.0-58.7]
Participants in COVID-19 research	856 (32.8) [29.2-36.7]	539 (26.3) [24.2-28.4]	1777 (89.7) [88.2-91.1]
Age groups			
Healthy adults aged ≥65 y	892 (35.6) [31.9-39.5]	545 (27.6) [25.5-29.9]	1582 (87.6) [85.8-89.1]
Healthy adults aged 30-65 y	NA	169 (8.6) [7.3-10.1]	945 (60.8) [58.2-63.4]
Young adults aged 18-29 y	NA	112 (5.5) [4.5-6.7]	976 (53.1) [50.6-55.6]
Healthy adults age 18-65	108 (5.1) [3.6-7.3]	NA	NA
Children aged 0-18 y	238 (13.1) [10.6-16.2]	NA	NA
Teenagers	NA	118 (6.7) [5.5-8.1]	1160 (60.0) [57.7-62.4]
Young children	NA	273 (14.2) [12.6-16.0]	1366 (69.5) [67.3-71.6]

Abbreviation: NA, not applicable.

Older respondents were less likely to prioritize healthy people older than 65 years for vaccination (eTable 1, eTable 2, and eTable 3 in the Supplement). Respondents in both surveys aged 65 years and older were significantly less likely than those younger than 65 years to rank healthy adults aged 65 and older among their 4 highest priority groups (Gallup, 23.7% vs 39.1% [χ^2^ = 2160.8; *P* < .001]; COVID Collaborative, 23.3% vs 28.8% [χ^2^ = 5.0198; *P* = .03]). There was substantial agreement between survey respondents’ highest priorities for vaccine distribution and NASEM’s and ACIP’s phased COVID-19 vaccine distribution plans (Table 3).

**Table 3.** NASEM Priority Groups and Public Preferences

NASEM phase	Respondents, % (95% CI)		
	Gallup (top 4 groups)	COVID collaborative	
		Top 4 groups	Willingness to wait behind
Phase 1a: health care workers providing direct patient care; first responders	Health care workers: 93.6 (91.2-95.3)	Health care workers: 80.0 (78.0-81.9)	Health care workers: 96.6 (95.6-97.4
Phase 1b: people of all ages with multiple high-risk conditions; older adults living in congregate or overcrowded settings	Medically vulnerable: 78.6 (75.2-81.7)	Medically vulnerable: 72.9 (70.7-74.9)	Nursing home residents: 96.2 (95.1-97.0)
		Nursing home residents: 55.0 (52.6-57.3)	Medically vulnerable: 95.6 (94.4-96.5)
Phase 2: essential workers facing high risk of SARS-CoV-2 exposure; teachers, school staff, and childcare workers; people of all ages with a single high-risk condition; all older adults not included in phase 1; people living and working in congregate settings such as homeless shelters and jails	Teachers and childcare workers: 48.3 (44.3-52.2)	Teachers and childcare workers: 32.2 (30.0-34.4)	Teachers and childcare workers: 92.5 (91.2-93.7)
	Other nonhealth essential workers: 47.1 (43.2-51.1)	Healthy adults aged ≥65 y: 27.6 (25.5-29.9)	Healthy adults aged ≥65 y: 87.6 (85.8-89.1)
	Adults in group settings: 45.7 (41.8-49.7)	Grocery store workers: 14.4 (12.8-16.2)	Grocery store workers: 85.9 (84.2-87.5)
	Healthy adults aged ≥65 y: 35.6 (31.9-39.5)	Prisoners and prison guards: 8.2 (6.9-9.6)	Prisoners and prison guards: 56.4 (54.0-58.7)
Phase 3: young adults, children, and essential workers not included in phases 1 or 2	Children aged <18 y: 13.1 (10.6-16.2)	Restaurant workers: 8.9 (7.7-10.4)	Restaurant workers: 74.2 (72.1-76.2)
		Young children: 14.2 (12.6-16.0)	Children: 69.5 (67.3-71.6)
		Teenagers: 6.7 (5.5-8.1)	Teens: 60.0 (57.7-62.4)
		Healthy adults aged 18-29 y: 5.5 (4.5-6.7)	Healthy adults aged 18-29 y: 53.1 (50.6-55.6)
Phase 4: everyone else	Healthy adults aged 30-65 y: 5.1 (3.6-7.3)	Healthy adults aged 30-65 y: 8.6 (7.3-10.1)	Healthy adults aged 30-65 y: 60.9 (58.2-63.4)

Abbreviation: NASEM, National Academies of Science, Engineering, and Medicine.

### Prioritization of Disproportionately Impacted Communities

Both Gallup COVID-19 Panel and COVID Collaborative respondents thought that racial/ethnic communities disproportionately impacted by COVID-19 should receive priority for a vaccine (eTable 1, eTable 2, and eTable 3 in the Supplement). Fully 74.2% (95% CI, 70.6%-77.5%) of Gallup COVID-19 Panel respondents concurred that “[m]embers of some groups (such as Black, Hispanic and Native American individuals) are at a much higher risk of getting sick with and dying from COVID-19. Should these groups have access to the COVID-19 vaccine before lower-risk groups?” Although Republicans were significantly less likely than Democrats to prioritize these groups, majorities from both political parties agreed (60.4% vs 87.7%; χ^2^ = 12.001; *P* < .001). Similarly, 84.9% (95% CI, 83.1%-86.5%) of COVID Collaborative respondents accepted vaccine prioritization for “Black people, Hispanics, and Native Americans, which are communities that have had higher rates of COVID-19.” Notably, 76.9% (95% CI, 71.7%-82.1%) of very conservative respondents accepted this prioritization, the lowest rate among political categories but still a supermajority (76.9% vs 86.4%; χ^2^ = 18.25; *P* < .001).

### Place in Line

Only COVID Collaborative respondents were asked to “indicate if [they] would be okay or not okay with people in [various] groups being allowed to get the COVID-19 vaccine *before* you can get it.” Majorities of respondents agreed to be preceded by 6 groups, including “healthcare workers” (96.6%; 95% CI, 95.6%-97.4%), “nursing home residents and staff” (96.2%; 95% CI, 95.1%-97.0%), “people with serious medical conditions that make them more likely to have complications or die from COVID-19” (95.6%; 95% CI, 94.4%-96.5%), “teachers and childcare workers” (92.5%; 95% CI, 91.2%-93.7%), “people who participated in research to find a safe and effective COVID-19 vaccine or…treatment” (89.7%; 95% CI, 88.2%-91.1%), “grocery store workers” (85.9%; 95% CI, 84.2%-87.5%), and “workers at restaurants, bars, and gyms” (74.2%; 95% CI, 72.1%-76.2%). Furthermore, a majority of respondents (56.4%; 95% CI, 54.0%-58.7%) approved of being preceded in line by “people in prisons and prison guards.” Very liberal respondents were likelier than others to accept people in prison and prison guards receiving the vaccine before them (67.7% vs 54.6%; χ^2^ = 16.178; *P* < .001). Respondents of color were significantly more likely than White respondents to accept being preceded by younger age groups, including children aged 12 years and younger (76.4% vs 65.5%; χ^2^ = 26.313; *P* < .001), teenagers aged 13 to 18 years (66.6% vs 56.2%; χ^2^ = 21.121; *P* < .001), young adults between the ages of 19 and 29 years (61.6% vs 48.4%; χ^2^ = 32.382; *P* < .001), and healthy adults between the ages of 30 and 65 years (70.0% vs 57.0%; χ^2^ = 19.559; *P* < .001).

### Considerations for Prioritization

Only COVID Collaborative respondents were asked to “select the four considerations you think should be most important” when deciding who should be vaccinated first. The 4 most commonly selected considerations were “focus on what will most prevent the spread of the virus” (78.4%; 95% CI, 76.3%-80.3%), “focus on what will prevent the most deaths” (72.1%; 95% CI, 69.9%-74.2%), “focus on what will protect the most people from long-term health complications” (68.9%; 95% CI, 66.6%-71.9%), and “focus on protecting the frontline workers” (63.8%; 95% CI, 61.5%-66.1%). Black, Hispanic, and Asian respondents were more likely than White respondents to select “focus on what will prevent the most lost years of life” (26.5% vs 20.0%; χ^2^ = 11.475; *P* < .001). Approximately one-third (36.5%; 95% CI, 34.2%-38.9%) of respondents ranked “focus on what will most help the economic recovery” as a top consideration. Very conservative respondents were significantly more likely than other respondents to endorse focusing on economic recovery (52.7% vs 33.3%; χ^2^ = 40.185; *P* < .001), as were Republican respondents (45.1% vs 25.1%; χ^2^ = 84.821; *P* < .001).

## Discussion

These 2 surveys involving nearly 5000 US adults reveal remarkable public consensus on controversial questions regarding COVID-19 vaccine allocation. Most respondents agreed that health care workers should be vaccinated first, followed by medically vulnerable people and nursing home residents and staff. The results suggest substantial community-mindedness, as more than 80% of respondents were also willing to wait in line behind teachers, grocery store workers, and people in Black, Hispanic, Native American, and other communities that have been disproportionately affected by COVID-19. Respondents prioritized these groups over healthy older adults. Respondents of color were likelier than White respondents to favor giving priority to younger individuals. The public’s views generally comport with NASEM’s and ACIP’s vaccine distribution recommendations, although the public places lower priority on vaccinating healthy older adults. Five points with policy relevance bear emphasis.

### Highest Priority Groups

First, across both surveys, most respondents prioritized health care workers for COVID-19 vaccination. Majorities of respondents also ranked nursing home residents and staff, as well as adults of any age with serious health conditions, among their highest priority groups. Our findings are consistent with those of a prior survey of 1007 respondents conducted by Gollust et al.^15^ Bipartisan agreement on these central prioritization decisions was notable.

Second, respondents assigned markedly less importance to prioritization on the basis of older age alone. The World Health Organization, former Secretary of Health and Human Services Alex Azar, and other prominent commentators have advocated for use of an age cutoff to define the highest priority group.^18^ Some states have adopted a similar approach. As of February 2021, Florida and South Carolina remained in phase 1a, offering vaccines to all people older than 65 years alongside health care workers and nursing home residents but before frontline workers or most people with serious medical conditions.^19^ A few states have removed all priority factors other than age, thereby ranking healthy people younger than 65 years ahead of even marginally younger frontline workers or medically vulnerable people. Survey respondents consistently ranked medically vulnerable people, nursing home residents, and adults living in group settings as higher priority groups for vaccination than healthy older adults. These findings suggest that the public prefers allocation approaches like ACIP’s and NASEM’s that honor multiple values, although such plans may require more effort to implement. Most states continue to base eligibility on multiple factors rather than solely or primarily on age, although many have opened eligibility for healthy adults aged 65 to 74 years earlier than recommended by the ACIP and/or have narrowed the eligibility requirements frontline worker.^19^

Importantly, older respondents were less likely to prioritize themselves for COVID-19 vaccine access. This departs sharply from prior findings,^15^ which indicated that older people were likelier to prioritize themselves for vaccination. The difference could reflect the fact that our questions—following NASEM’s language—distinguished healthy adults older than 65 years from medically vulnerable adults and those in nursing homes. Consistent with other studies, we found that respondents of color were likelier than White respondents to accept priority for young children, teens, and adults younger than 65 years. Recent findings that middle-aged adults face substantial risk from COVID-19 and that risk for younger and middle-aged patients is disproportionately higher in Black, Hispanic, Native American, Pacific Islander, and Asian American communities make this difference particularly relevant to the acceptability of age cutoffs in allocation policies.^20,21,22,23^ Notably, although these surveys were conducted before COVID-19 vaccine authorization, the Food and Drug Administration has authorized emergency use of the Pfizer-BioNTech COVID-19 vaccine only for individuals aged 16 years and older and of the Moderna COVID-19 vaccine for individuals aged 18 years and older. Our findings underscore the importance of ongoing research to determine the safety and efficacy of COVID-19 vaccines for children and teenagers, so that vaccines are eventually available to them.^24^

Respondents agreed with many of NASEM’s occupational rankings, giving highest priority to teachers and childcare workers, then to grocery store workers, and finally to workers at restaurants, bars, and gyms. Notably, more respondents endorsed prioritization of teachers and childcare workers than healthy adults aged 65 years or older or grocery store workers, which has implications for their relative prioritization within NASEM’s phase 2 and ACIP’s phase 1b.^3,25^ This preference aligns with ethical arguments that reopening schools and allowing undisrupted learning without parental supervision is important for equity and benefit to children and their families and conflicts with recent state choices to rank teachers and other childcare workers behind healthy older adults.^26^

### Prioritization to Address Health Inequities

Third, given the national discussion—and sometimes disagreement—about systemic racism and its relevance to the COVID-19 pandemic,^27^ it is noteworthy that sizeable majorities of respondents across all political affiliations endorsed prioritizing groups who have experienced worse outcomes from COVID-19, explicitly including people of color, for vaccination. Given the disparities in harm wrought by COVID-19, NASEM has proposed to address health inequities in prioritizing vaccination.^2^ Similarly, President Biden’s National Strategy for the COVID-19 Response and Pandemic Preparedness commits to “driv[ing] equity in vaccinations by using demographic data to identify communities hardest hit by the virus and … making sure vaccines reach those communities.”^28^ Rhode Island elected to focus early vaccine access on one such community,^29^ and other states and localities have used zip codes or vulnerability indices to prioritize vaccine distribution.^30,31^ Our findings suggest that US adults endorse vaccine allocation policies and implementation strategies that achieve priority access for Black, Hispanic, Native American, and other communities who have experienced high numbers of COVID-19 cases, as well as more and earlier deaths. They also imply that an important part of messaging will be to emphasize the COVID-19 burden borne by these groups as the reason for their prioritization. Importantly, policies that explicitly recognize and address population-level racial disparities without classifying individual beneficiaries by race have been upheld in court in other contexts^32^ and have been implemented and upheld for other aspects of COVID-19 response.^33,34^

Whether prisoners should be given priority for vaccination has also been prominently discussed because prisons are high-risk settings for COVID-19 spread.^30^ Some states, such as Massachusetts, have given prisoners priority whereas others, such as Colorado, have not.^35^ NASEM placed prisoners and prison guards in phase 2, along with teachers, grocery store workers, and healthy older adults. A majority of survey respondents accept some degree of priority for prisoners, disagreeing with Colorado’s denial of priority. However, respondents appear less willing to prioritize prisoners and prison guards than other phase 2 groups. This may indicate that people convicted of crimes are perceived as less deserving of COVID-19 vaccines than others at similar risk, paralleling survey responses in other contexts.^36,37^

Fourth, respondents’ answers could also inform current debates over whether COVID-19 vaccine trial participants who received placebos should now be prioritized for vaccination.^38^ Although COVID Collaborative respondents were comfortable with people who participated in COVID-19 research preceding them in line, only one-quarter ranked research participants among their 4 highest-priority groups. This suggests potential public support for an intermediate approach, such as allowing trial participants to precede others within the same group (eg, among essential workers).^39^

### Considerations for Prioritization

Fifth, respondents’ top considerations for prioritization largely tracked NASEM’s ethical framework, rather than a single-principle approach of maximizing near-term lives saved directly by vaccination. Preventing spread of the virus and preventing death aligns with the principle of maximum benefit. Protecting frontline workers—who are likelier to be lower-paid and members of groups subject to structural discrimination—and disproportionately impacted communities aligns with mitigation of health inequities.^2^ Vaccinating health care workers, a goal respondents prioritized, may be seen as instrumental to achieving other goals, such as preventing deaths and spread of the virus. Like NASEM, respondents placed low importance on economic benefits. Many respondents also regarded preventing long-term health impacts as very important. This diverges from NASEM’s proposal and from many state plans, which have emphasized reducing the number of deaths and accordingly prioritized groups at high short-term mortality risk. Finally, respondents of color were likelier to regard prioritizing recipients with more years left to live as important, aligning with their stronger preferences for prioritizing children and young adults. This may reflect the greater proportion of COVID-19 deaths among younger and middle-aged people in these communities of color^23,40,41^ or differences in values.^7^

### Limitations

Generalizing survey results to the population of interest is based on the assumption that respondents are a representative sample of the population; given our response rates, it is possible that there was nonresponse error and our survey estimates may be biased. Conducting the survey in English omitted some segments of the US population. In addition to sampling error, question wording and difficulties in conducting online surveys can introduce error and bias into the findings of public opinion polls. However, for those questions where there was overlap, finding consistent responses across the 2 surveys despite different wordings and different sampling methods suggests that potential errors are less likely in this pair of surveys. Given the sensitive nature of allocation decisions, social desirability bias may have affected respondents’ answers. In both surveys, questions related to priority for Black, Hispanic, and Native American communities noted that members of these communities are at “higher risk of getting sick with and dying from COVID-19” or have “higher rates of COVID-19.” This framing may have increased concurrence with the statement. The surveys were fielded before issuance of emergency use authorizations for the Pfizer-BioNTech and Moderna vaccines; thus, survey respondents were asked about distribution of a hypothetical COVID-19 vaccine. Responses may differ now that COVID-19 vaccines are available and the practical implications of limited doses and phased distribution are becoming clear.

## Conclusions

The findings of these 2 surveys of US adults suggest that members of the US public agreed with core elements of NASEM’s and ACIP’s COVID-19 phased vaccine distribution plans. They also endorsed prioritizing disproportionately affected communities, including communities of color. Respondents differed with government bodies and officials regarding purely age-based prioritization and strongly endorsed priority for teachers and childcare workers. Particularly noteworthy was older respondents’ lesser support for prioritizing healthy people older than 65 years and respondents’ greater support for prioritizing younger and middle-aged recipients. These findings indicate that the public would be supportive of prioritization approaches that effectively recognize multiple values, rather than basing allocation solely on age or any other single factor. Policy makers should build on existing efforts, such as proactive outreach to vulnerable communities and workplaces, and commit to investing resources to achieve vaccine distribution that is both speedy and consonant with public values.

## Notes

Supplement. **eAppendix 1.** Gallup Survey **eAppendix 2.** COVID Collaborative Survey **eTable 1.** Gallup Survey vs COVID Collaborative Survey **eTable 2.** Support for Prioritizing Communities Hard Hit by COVID-19 **eTable 3.** Reasons for Prioritization (Select 2)
