# Supplementary material for: Public Perspectives on COVID-19 Vaccine Prioritization
Source: JAMA Netw Open. 2021 Apr 9;4(4):e217943. doi: 10.1001/jamanetworkopen.2021.7943 (PMC8035644; doi:10.1001/jamanetworkopen.2021.7943)
Supplement: Supplement. — eAppendix 1. Gallup Survey eAppendix 2. COVID Collaborative Survey eTable 1. Gallup Survey vs COVID Collaborative Survey eTable 2. Support for Prioritizing Communities Hard Hit by COVID-19 eTable 3. Reasons for Prioritization (Select 2) [file jamanetwopen-e217943-s001.pdf]

Supplemental Online Content

Persad G, Emanuel EJ, Sangenito S, Glickman A, Phillips S, Largent EA. Public perspectives on COVID-19 vaccine prioritization. *JAMA Netw Open*. 2021;4(4):e217943. doi:10.1001/jamanetworkopen.2021.7943

eAppendix 1. Gallup Survey
eAppendix 2. COVID Collaborative Survey
eTable 1. Gallup Survey vs COVID Collaborative Survey
eTable 2. Support for Prioritizing Communities Hard Hit by COVID-19
eTable 3. Reasons for Prioritization (Select 2)

This supplemental material has been provided by the authors to give readers additional information about their work.

© 2021 Persad G et al. *JAMA Network Open.*

eAppendix 1. Gallup Survey

INTRO If there is a COVID-19 vaccine in 2021 it will be in short supply, and there will not be enough doses for everyone to get it quickly. We want to ask your views on who should have access to the COVID-19 vaccine first.

Q01 Please rank the following groups in the order that you feel they should have access to the COVID-19 vaccine. *(For example, the group you select as 1st would be the first to get the vaccine.)*
(PROGRAMMER: RANDOMIZE Q01A – Q01I)

1 1st
2 2nd
3 3rd
4 4th
5 5th
6 6th
7 7th
8 8th
9 9th

Q01A Healthcare workers
Q01B Adults of any age with serious health conditions
Q01C Healthy adults age 65 or older
Q01D People who participated in COVID-19 vaccine research
Q01E Children under the age of 18
Q01F Elementary school teachers and staff
Q01G Non-healthcare essential workers (such as grocery store, agriculture, or childcare workers)
Q01H Healthy adults under the age of 65
Q01I Adults living in group settings

Q02 Which of the following reasons best describe how you decided to order access to the COVID-19 vaccine? *You may select up to two responses.*
(PROGRAMMER: ROTATE 1-9)

1 Preventing the most deaths
2 Protecting those with more years to live
3 Preventing deaths of young people
4 Preventing COVID-19 in groups more likely to have health problems
5 Making sure essential workers can stay on the job
6 Rewarding workers who have taken risks to help others during COVID-19
7 Protecting those who will contribute the most to the economy
8 Protecting those who are most likely to die from COVID-19 if they catch it
9 Protecting those whose professions increase their chances of being in contact with someone with COVID-19
10 Preventing COVID-19 in people who are most likely to spread it to others
Other

© 2021 Persad G et al. *JAMA Network Open.*

Q06 Members of some groups (such as Black, Hispanic and Native American individuals) are at a much higher risk of getting sick with and dying from COVID-19. Should these groups have access to the COVID-19 vaccine before lower-risk groups?

1 Yes
2 No

Q03 Some companies are suggesting that they will **require** workers to get the COVID-19 vaccine if they want to return to work (unless they have a medical reason to not get vaccinated). How acceptable would it be for companies to require workers to get the COVID-19 vaccine?

1 Very acceptable
2 Acceptable
3 Neither acceptable nor unacceptable
4 Unacceptable
5 Very unacceptable

Q04 Some states are suggesting that they will **require** adults in that state to get the COVID-19 vaccine (unless they have a medical reason to not get vaccinated). How acceptable would it be for states to require adults to get the COVID-19 vaccine?

1 Very acceptable
2 Acceptable
3 Neither acceptable nor unacceptable
4 Unacceptable
5 Very unacceptable

Q05 Some states are suggesting that they will **require** children to get the COVID-19 vaccine as a condition for attending school (unless they have a medical reason to not get vaccinated). How acceptable would it be for states to require children to get the COVID-19 vaccine?

1 Very acceptable
2 Acceptable
3 Neither acceptable nor unacceptable
4 Unacceptable
5 Very unacceptable

© 2021 Persad G et al. *JAMA Network Open.*

eAppendix 2. COVID Collaborative Survey

Hart Research Associates, a national public opinion polling firm, is conducting a survey about issues facing the country, and we would really appreciate the chance to get your opinions on a few questions. This is a public opinion survey, and you will not be sold anything. Your name and individual opinions will be kept confidential. Responses will only be reported in the aggregate.

FORM Which FORM is this? (DO NOT SHOW.)

237FORM A	1	108
236FORM B	2	

QS1 We care about the quality of our data. In order for us to get the most accurate measures of your knowledge and opinions, it is important that you read all questions carefully, thoughtfully provide your best answers to each question in this survey, and type your responses in as much detail as possible.

Do you commit to thoughtfully providing your best answers to each question in this survey? 109

105

I will provide my best answers.	1	**CONTINUE**
I will not provide my best answers.	2	**TERMINATE**
I can't promise either way.	3	**TERMINATE**

QS2 For statistical purposes only, please indicate how old you are.

106-107

Under 18	0	**TERMINATE**
18-24	1	110-111
25-29	2	
30-34	3	
35-39	4	
40-44	5	
45-49	6	**CONTINUE**
50-54	7	
55-59	8	
60-64	9	
65-69	10	
70-74	11	
75 or older	12	

QS3 In which state do you currently live? **(DROP-DOWN MENU OF ALL STATES AND D.C.) 112-117 333-334**
108-109

QS4 Are you…?

110

Male	1	118
Female	2	
Non-binary/other	3	

QS5a Are you of Hispanic, Latino, or Spanish origin?

111

Yes, Hispanic/Latino/Spanish origin	1	119
No, not Hispanic/Latino/Spanish origin	2	

QS5b Please indicate your race.

112

White	1	120
Black/African American	2	
Asian	3	
Native American/American Indian	4	
Other	5	

© 2021 Persad G et al. *JAMA Network Open.*

(ASK EVERYONE.)

QS5abTH Are you of Hispanic, Latino, or Spanish origin? Please indicate your race.

113White	1	121
114Black/African American	2	
115Asian	3	
116Native American/American Indian	4	
117Hispanic		
118Other	5	

(ASK EVERYONE.)

QS6a What is the last grade that you completed in school?
119

Grade school or less	1	122
Some high school	2	
High school graduate	3	
Some college, no degree	4	
Vocational training/2-year college	5	
4-year college/bachelor's degree	6	
Some postgraduate work, no degree	7	
2 or 3 years' postgraduate work/master's degree	8	
PhD or professional degree (MD, JD, MBA)	9	

QS6b What is the best way to describe the area where you live?
120

A large city	1	123
A small city	2	
A suburb near a city	3	
A small town	4	
A rural area	5	

QS7 Generally speaking, do you think of yourself as a…? **(ALTERNATE ORDER OF PUNCHES 1-5, 5-1, WITH "COMPLETELY INDEPENDENT" ALWAYS PUNCH 3.)**
121

Strong Republican	1	124
......		
Leaning Republican	2	
Completely independent	3	
Leaning Democrat	4	
Strong Democrat	5	

QS8a Which of the following best describes your current employment status? 125
122

Employed full time	1	**CONTINUE**
Employed part time (less than 35 hours per week)	2	**CONTINUE**
Unemployed but looking for work	3	**CONTINUE**
Full-time student	4	**SKIP TO MATH QC**
Stay-at-home parent	5	**SKIP TO MATH QC**
Retired	6	**SKIP TO MATH QC**

(ASK ONLY OF RESPONDENTS WHO SAY "EMPLOYED" OR "UNEMPLOYED" IN QS8a.)

QS8b Have you either lost your job or experienced a cut in your pay or wages because of the situation with the coronavirus?
711

Yes, I have lost my job or experienced a cut in pay because of the

© 2021 Persad G et al. *JAMA Network Open.*

coronavirus. 1 126
....
No, I have not lost my job or experienced a cut in pay because of the coronavirus. 2

ADD MATH QUALITY CONTROL QS

Q1 Do you feel that the country is moving in the right direction or the wrong direction these days? (13024-Q1)
142

Country is moving in the right direction.. 1 127
....
Country is moving in the wrong direction... 2

Q2 In terms of the spread of the coronavirus, do you think the worst is behind us or the worst is still ahead of us?
712

The worst is behind us, and the spread of the coronavirus is no longer a serious problem. 1 128
The worst is behind us, but the spread of the coronavirus is still a serious problem. 2
The worst is still ahead of us. 3

Q3 How much do you worry that you or someone in your immediate family might get sick with the coronavirus, also known as COVID-19?
143

Worry a lot 1 129
Worry some 2
Worry just a little 3
Do not worry at all.. 4

Q4 When you think about the overall health of your community, do you think the coronavirus, also known as COVID-19, is…?
144

No more dangerous than the seasonal flu... 1 130
Somewhat more dangerous than the seasonal flu 2
Much more dangerous than the seasonal flu.. 3

(SHOW TO EVERYONE ON A SEPARATE SCREEN.)
For the rest of this survey, we will refer to COVID-19, which is another name for the coronavirus that is currently affecting the United States and other countries around the world.

Q5 How important do you feel it is for the United States to have a vaccine for COVID-19?
145

Very important 1 131
Fairly important 2
Only somewhat important 3
Not that important 4
Not important at all 5

Q6a Please rank, in order of importance, the steps below that you think will have the biggest impact on controlling the spread of COVID-19. **(RANDOMIZE ITEMS. SET UP AS DRAG-AND-DROP. REQUIRE RESPONDENT TO RANK THREE.)**

1. 713 People wearing masks
2. 714 People getting vaccinated for COVID-19 when a vaccine is available
3. 715 People practicing social distancing (e.g., keeping six feet apart from other people)
4. 716 Having a strong system for COVID-19 testing
5. 717 Having a strong system for contact tracing

© 2021 Persad G et al. *JAMA Network Open.*

6. 718 Having nonessential employees work from home
7. 719 Having schools open at a reduced capacity, either with all or some students learning remotely

1st most important	_____	132
2nd most important	_____	133
3rd most important	_____	134

Q6b If you were interested in getting a COVID-19 test, which of the following factors would be most important to you? **(RANDOMIZE ITEMS. SET UP AS DRAG-AND-DROP. REQUIRE RESPONDENT TO RANK THREE.)**

1. 720 The test is free
2. 721 A rapid test with results available within 15 minutes
3. 722 A saliva test
4. 723 A test that involves a shallow nose swab
5. 724 A test with highly reliable results (80% accuracy or better)
6. 725 A test you could take at home and get the result at home

1st most important	_____	135
2nd most important	_____	136
3rd most important	_____	137

(SHOW TO EVERYONE ON A SEPARATE SCREEN.)
Thinking more about a COVID-19 vaccine…

Q7a How likely are you to get vaccinated for COVID-19 when a vaccine becomes available for you?
726

I definitely will get vaccinated.	1	138
I probably will get vaccinated.	2	
I probably will NOT get vaccinated.	3	
I definitely will NOT get vaccinated.	4	
I am completely undecided about whether I will get vaccinated.	5	

Q7b Thinking about this a different way, which of the following statements comes closest to what you are most likely to do when a COVID-19 vaccine becomes available for you?
727

I will get vaccinated as soon as possible.	1	139
I will wait to see what happens to other people who are vaccinated before deciding whether to get vaccinated myself.	2	
I will not get vaccinated, regardless of what happens to other people who get the vaccine.	3	

Q8 How important do you think it is that a majority of Americans eventually get vaccinated for COVID-19?
728

Very important	1	140
Fairly important	2	
Only somewhat important	3	
Not that important	4	
Not important at all	5	

(ASK EVERYONE.)
Q10a Over the past five years, how often have you gotten a flu shot?
146

Every year	1	141
Most years	2	
Only some years	3	
Never	4	

© 2021 Persad G et al. *JAMA Network Open.*

Q10b How likely is it that you will get a flu shot this fall or winter?
147

I have already gotten a flu shot. 1 142
I definitely will get a flu shot. 2
I probably will get a flu shot. 3
I probably will NOT get a flu shot. 4
I definitely will NOT get a flu shot. 5

Q11a Do you have children under the age of 18 living with you in your household? If, so please indicate the ages of your child(ren). *Select all ages that apply.* **(ACCEPT MULTIPLE RESPONSES.)**

729Yes, child/children under age 2 .... 1 143
730Yes, child/children age 2 to 5............ 2 **CONTINUE**
731Yes, child/children age 6 to 12............ 3
732Yes, child/children age 13 to 17............ 4
733No, there are no children under the age of 18 in my household .... 5 **SINGLE PUNCH, SKIP TO Q12**

(ASK ONLY OF RESPONDENTS WHO HAVE CHILD/CHILDREN IN HOUSEHOLD IN Q11a (p1:4).)

Q11b As you know, the Centers for Disease Control and most physicians recommend that children get vaccinated for a variety of diseases and illnesses, such as polio, measles, whooping cough and flu. Some parents and guardians choose to have their children get these vaccinations, but other parents and guardians do not.

Have the children in your household gotten all of these recommended vaccines for their age, some of them, or none of them?
734

All of these recommended vaccines 1 144
Some of these recommended vaccines............ 2
None of these recommended vaccines 3

(ASK ONLY OF RESPONDENTS WHO HAVE CHILD/CHILDREN IN HOUSEHOLD IN Q11a (p1:4) AND SAY THEIR CHILDREN HAVE SOME OR NONE OF RECOMMENDED VACCINES IN Q11b (p2:3).)

Q11c Which statement best describes why the children in your household have not gotten all of the recommended vaccines for their age?
735

My child/children cannot get vaccines for medical reasons. 1 145
....
I do not think the vaccines are necessary. 2
I do not think the vaccines are safe. 3

Q13a AND Q13b ARE ROTATED.

Q13a When a COVID-19 vaccine is approved by the U.S. Food and Drug Administration as safe and effective, how confident are you that it will actually be safe, with no harmful side effects for people who get the vaccine?
148

Very confident............ 1 146
Somewhat confident 2
Not that confident 3
Not confident at all............ 4

Q13b When a COVID-19 vaccine is approved by the U.S. Food and Drug Administration as safe and effective, how confident are you that it will actually be effective and prevent most people who get the vaccine from getting COVID-19?
149

Very confident............ 1 147
Somewhat confident 2
Not that confident 3
Not confident at all............ 4

© 2021 Persad G et al. *JAMA Network Open.*

Q14a Compared with other situations when the U.S. Food and Drug Administration approves a medical treatment as being safe and effective, are you more confident or less confident in the approval process for a COVID-19 vaccine?148

736

More confident in the approval process for a COVID-19 vaccine .... 1 **CONTINUE**
Less confident in the approval process for a COVID-19 vaccine............ 2 **CONTINUE**
About the same level of confidence as for other medical treatments that are approved by the FDA... 3 **SKIP TO Q15a**

(ASK ONLY OF RESPONDENTS WHO SAY "MORE CONFIDENT" OR "LESS CONFIDENT" IN Q14a (P1:2).)

Q14b Are there any specific reasons why you are (more/less) confident in the FDA approval process for a COVID-19 vaccine, compared with other medical treatments approved by the FDA? If so, please explain. If not, please indicate that. **(RESPONSE REQUIRED.)** **149-159**

(ASK EVERYONE.)

Q15a When a COVID-19 vaccine is approved as being safe and effective, do you think it will be available to most people almost right away or that most people will have to wait before they can get vaccinated for COVID-19? 740 160

Will be available for most people almost right away .... 1 **SKIP TO Q16a/b**
Most people will have to wait before they can get it............................... 2 **CONTINUE**

(ASK ONLY OF RESPONDENTS WHO SAY "MOST PEOPLE WILL HAVE TO WAIT" IN Q15a (P2).)

Q15b How long do you think it will take before most people who want to be vaccinated will be able to get the COVID-19 vaccine, once it is approved?

741

One or two months 1 161
Three to six months 2
Seven to twelve months 3
More than a year 4

(FORM A)

Q16a When a safe and effective COVID-19 vaccine is widely available, do you agree or disagree that <u>all Americans should be expected to get the COVID-19 vaccine, unless they have a clear medical reason not to get the vaccine</u>?

742

Strongly agree 1 162
Somewhat agree 2
Somewhat disagree.. 3
Strongly disagree... 4

(FORM B)

Q16b When a safe and effective COVID-19 vaccine is widely available, do you agree or disagree that <u>all Americans have a responsibility to get the COVID-19 vaccine, unless they have a clear medical reason not to get the vaccine</u>?

743

Strongly agree 1 163
Somewhat agree 2
Somewhat disagree.. 3
Strongly disagree... 4

(ASK EVERYONE.)

Q16abTH When a safe and effective COVID-19 vaccine is widely available, do you agree or disagree that <u>all Americans (**FORM A**: should be expected)(**FORM B**: have a responsibility) to get the COVID-19 vaccine, unless they have a clear medical reason not to get the vaccine</u>?

© 2021 Persad G et al. *JAMA Network Open.*

744Strongly agree 1 164
745Somewhat agree 2
746Somewhat disagree 3
747Strongly disagree 4

(ASK EVERYONE.)

Q16c If someone opts NOT to get the COVID-19 vaccine when it is available to them, and does not have a specific health reason not to do so, would your view of that person be…?

749

Favorable 1 165
Unfavorable 2
Neither favorable nor unfavorable 3

(ASK EVERYONE.)

Q16d Suppose most Americans get a COVID-19 vaccine after it becomes available. How effective do you think that would be in reducing the spread of COVID-19?

750

Very effective 1 166
Somewhat effective 2
Not that effective 3
Not effective at all 4

Q17 AND Q18 ARE ROTATED.

Q17 Below are reasons that people have given for why they will get the COVID-19 vaccine. Please indicate how important a motivation each one is to you personally for getting the COVID-19 vaccine, using a scale from 0 to 10. **(RANDOMIZE ITEMS. USE MOBILE-FRIENDLY BUTTON FORMAT.)**

1. 768-769To protect myself 167-168
2. 787-788(**ASK ONLY OF RESPONDENTS WHO SAY THEY HAVE CHILDREN IN Q11a)** To protect my children 169-170
3. 806-807To protect elderly people in my family 171-172
4. 825-826-To protect a friend or family member who is particularly vulnerable to COVID-19 because they have an underlying medical condition 173-174
5. 844-845To protect the health of people in my community 175-176
6. 863-864-To allow the economy to fully reopen and get back to normal 177-178

Not important motivation at all									Extremely important motivation	
0	1	2	3	4	5	6	7	8	9	10

Q18 Below are some reasons people may give for why they will NOT get the COVID-19 vaccine. For each one, please indicate whether that definitely applies to you, somewhat applies to you, or does not apply to you as a reason for not getting the COVID-19 vaccine. **(RANDOMIZE ITEMS.)**

1. 150I have a health condition that would make it risky for me to get the vaccine. 179
2. 151I have a right not to get vaccinated and choose to exercise that right. 180
3. 865I do not trust vaccines in general. 208
4. 866I do not think the vaccine will be effective in protecting me from COVID-19. 209
5. 867I do not think the vaccine will be safe and could have harmful side effects. 210

© 2021 Persad G et al. *JAMA Network Open.*

6. 868I am concerned approval of the vaccine will be rushed for political reasons, before adequate testing for safety and effectiveness is complete. 211
7. 869I do not think it is necessary to get vaccinated because I don't think COVID-19 is as big of a problem as it is being made out to be. 212
8. 870I do not think it is necessary to get vaccinated because I take precautions to protect myself, such as wearing a mask, maintaining a physical distance, and sanitizing. 213
9. 871I do not think I will get very sick if I get COVID-19. 214

Definitely applies to me 1
Somewhat applies to me 2
Does not apply to me.. 3

Q19 Below is a list of people and organizations that could make recommendations about getting the COVID-19 vaccine. Please indicate how much you trust each one when it comes to their recommendation about getting the COVID-19 vaccine. **(RANDOMIZE ITEMS.)**

(ALWAYS ASK FIRST)

1. 152 Donald Trump 215
2. 153 Joe Biden 216

(ALWAYS ASK SECOND)

3. 872 Dr. Anthony Fauci, Director of the National Institute of Allergy and Infectious Diseases 217
4. 873 The Centers for Disease Control and Prevention (CDC) 218
5. 874 The U.S. Surgeon General, who is the head of the nation's public health service 219
6. 875 The Food and Drug Administration (FDA) 220
7. 876 A bipartisan group of former Food and Drug Administration (FDA) Commissioners from previous Democratic and Republican administrations 221
8. 877 The governor of your state **(DO NOT ASK D.C. RESIDENTS) 222**
9. 878 Your own doctor 223
10. 879 Your own pharmacist 224
11. 880 Public health experts at a university in your state 225
12. 881 A friend you think of as knowledgeable and well-informed 226
13. 882 Your employer 227
14. 883 Your faith leader 228

Trust a lot 1
Trust somewhat.. 2
Trust just a little 3
Do not trust at all 4
I am not familiar with that individual or group.. 5

Q20 Recently, the CEOs of nine major pharmaceutical companies that are working to develop a COVID-19 vaccine pledged their commitment to developing and testing potential COVID-19 vaccines in accordance with high ethical standards and sound scientific principles. They pledged to continue to make safety and the well-being of vaccinated individuals the top priority in development of the first COVID-19 vaccines and to only submit a vaccine for approval after demonstrating safety and efficacy through the final stages of a clinical study.

How much do you trust the pharmaceutical companies to actually make safety and the well-being of vaccinated individuals the top priority in development of the first COVID-19 vaccine?

884

Trust a lot 1 229

© 2021 Persad G et al. *JAMA Network Open.*

Trust somewhat 2
Trust just a little 3
Do not trust at all 4

(ASK ONLY OF RESPONDENTS WHO SAY THEY PROBABLY WILL GET VACCINATED, PROBABLY WILL NOT, DEFINITELY WILL NOT, OR ARE UNDECIDED IN Q7a (P2:5).)

Q21a Below are some things that have been suggested to encourage more adults to get the COVID-19 vaccine. Please indicate whether each one would make you more likely to get vaccinated or not. **(RANDOMIZE ITEMS.)**

1. 885If it is available from a mobile clinic that comes to a location near your neighborhood (e.g., a mobile clinic that parks near a park, place of worship, or shopping center) 230
2. 886If you could receive it in your home from a nurse that goes door-to-door administering the vaccine. 231
3. **887(ASK ONLY OF RESPONDENTS WHO ARE EMPLOYED IN QS8a (P1:2))** If your employer required you to get vaccinated in order to go to work 232
4. 888If you had to pay a penalty to your state government for not getting vaccinated 233
5. 889If the vaccine were free 234
6. 890 If you were able to speak to a community health worker about the risks and benefits of the vaccine 235
7. 891 If you heard from top scientists and physicians that getting vaccinated is the most effective way to protect yourself and stop the spread of COVID-19 236
8. 892 If people like you (similar in age and demographic background) report a positive experience after taking the vaccine 237

Much more likely to get vaccinated 1
Somewhat more likely to get vaccinated 2
No more likely to get vaccinated 3

(FORM A)

Q21b If a COVID-19 vaccine were approved <u>before</u> the presidential election in November, would that give you more confidence in its safety and effectiveness, less confidence in its safety and effectiveness, or would it have no impact on your confidence either way?

154

Would make me more confident in its safety and effectiveness 1 238
Would make me less confident in its safety and effectiveness 2
Would have no impact on my confidence either way 3

(FORM B)

Q21c If a COVID-19 vaccine were approved <u>after</u> the presidential election in November, would that give you more confidence in its safety and effectiveness, less confidence in its safety and effectiveness, or would it have no impact on your confidence either way?

155

Would make me more confident in its safety and effectiveness 1 239
Would make me less confident in its safety and effectiveness 2
Would have no impact on my confidence either way 3

(ASK EVERYONE.)

Q22a When a COVID-19 vaccine is approved as safe and effective, it will take time for enough vaccines to be produced for everyone who wants to get vaccinated. That means federal and state officials will have to make decisions about who should get the vaccine first.

Below are different considerations that might affect decisions about who should get the vaccine first. Please select the four considerations you think should be most important in making these decisions. **(RANDOMIZE. SET UP AS DRAG-AND-DROP. REQUIRE RESPONDENT TO RANK FOUR.)**

1. 893 Focus on what will prevent the most deaths

© 2021 Persad G et al. *JAMA Network Open.*

2. 894 Focus on protecting younger people who have the most years left of life to lose
3. 895 Focus on what will most help the economic recovery
4. 896 Focus on what will most prevent the spread of the virus
5. 897 Focus on what will protect the most people from long-term health complications
6. 898 Focus on rewarding and protecting those people who most put themselves at risk during the pandemic--frontline workers
7. 899 Focus on protecting communities that have had the highest rates of COVID-19, including Black people, Hispanic people, and Native Americans

1st most important consideration	_____	240
2nd most important consideration	_____	241
3rd most important consideration	_____	242
4th most important consideration	_____	243

Q22b Which approach do you think federal and state officials should use when it comes to decisions about who should get the COVID-19 vaccine first? **(ROTATE.)**

900

A lottery system that would give everyone an equal chance to get the vaccine first..1 244

A priority system in which some groups would be able to get the vaccine first based on the considerations outlined in the previous question......2

Q23a Understanding that there will not be enough doses of the COVID-19 vaccine for everyone in the United States to get it immediately, please indicate whether you would be okay or not okay with people in each of the following groups being allowed to get the COVID-19 vaccine <u>before</u> you can get it. For any group that you are a part of, please indicate that. **(RANDOMIZE ITEMS.)**

1. 901 Children age 12 and younger 245
2. 902 Teenagers age 13 to 18 246
3. 903 Young adults between the ages of 19 and 29 247
4. 904 Healthy adults between the ages of 30 and 65 248
5. 905 Healthy adults age 65 and over 249
6. 906 People with serious medical conditions that make them more likely to have complications or die from COVID-19 250
7. 907 Teachers and childcare workers 251
8. 908 Healthcare workers (doctors, nurses, EMTs, and hospital staff) 252
9. 909 People in prisons and prison guards 253
10. 910 Nursing home residents and staff 254
11. 911 Grocery store workers 255
12. 912 Workers at restaurants, bars and gyms 256
13. 913 Communities that have had higher rates of COVID-19, including Black people, Hispanic people, and Native Americans 257
14. 914 People who participated in research to find a safe and effective COVID-19 vaccine or an effective treatment 258

Definitely okay with them getting the vaccine before I can 1
Probably okay with them getting the vaccine before I can......................2
Probably NOT okay with them getting the vaccine before can 3
Definitely NOT okay with them getting the vaccine before I can.............4
I am in this group...5

© 2021 Persad G et al. *JAMA Network Open.*

Q23b Still understanding that there will not be enough doses of the COVID-19 vaccine for everyone in the United States to get it immediately, please indicate which four of the following groups you think should be given the highest priority to get vaccinated before others. **(RANDOMIZE. SET UP AS DRAG-AND-DROP. REQUIRE RESPONDENT TO RANK FOUR.)**

1. 915 Children age 12 and younger 259-260
2. 916 Teenagers age 13 to 18 261-262
3. 917 Young adults between the ages of 19 and 29 263-264
4. 918 Healthy adults between the ages of 30 and 65 265-266
5. 919 Healthy adults age 65 and over
6. 920 People with serious medical conditions that make them more likely to have complications or die from COVID-19
7. 921 Teachers and childcare workers
8. 922 Healthcare workers (doctors, nurses, EMTs, and hospital staff)
9. 923 People in prisons and prison guards
10. 924 Nursing home residents and staff
11. 925 Grocery store workers
12. 926 Workers at restaurants, bars and gyms
13. 927 Communities that have had higher rates of COVID-19, including Black people, Hispanic people, and Native Americans
14. 928 People who participated in research to find a safe and effective COVID-19 vaccine or an effective treatment

1st highest priority group _____
2nd highest priority group _____
3rd highest priority group _____
4th highest priority group _____

Q24a Suppose there were a phased plan for the distribution of the COVID-19 vaccine in your state. The vaccine would be available in four different phases, starting with Phase 1 and ending with Phase 4. The people eligible to get the vaccine in each stage are listed below.

Phase 1 (first phase)--eligible groups:
High-risk workers in health facilities
First responders
People with preexisting conditions that put them at high risk
Elderly people living in facilities together

Phase 2--eligible groups:
Essential workers at high risk of exposure
Teachers and school staff
People with preexisting conditions of all ages with moderate risk
All older adults not included in Phase 1
People in homeless shelters or substance abuse recovery programs
People and staff in prisons

Phase 3--eligible groups:
Young adults
Children
Workers essential to society at increased risk of exposure and not included in phase 1 or 2

Phase 4 (last phase)--eligible groups:
Everyone in the state

© 2021 Persad G et al. *JAMA Network Open.*

Please indicate whether you support or oppose your state adopting this phased plan for the distribution of the COVID-19 vaccine. 267

156

Strongly support	1	**SKIP TO Q25a/b**
Somewhat support	2	**SKIP TO Q25a/b**
Somewhat oppose	3	**CONTINUE**
Strongly oppose	4	**CONTINUE**

(ASK ONLY OF RESPONDENTS WHO SAY "STRONGLY OR SOMEWHAT OPPOSE" IN Q24a (P3:4).)

Q24b What would your state need to change about this phased approach for the distribution of the COVID-19 vaccine for you to support it? Please be as specific and expansive as possible with your response. **(RESPONSE REQUIRED.)**

The phased plan is shown again below for your reference. 268-278

SHOW PHASED PLAN

© 2021 Persad G et al. *JAMA Network Open.*

Q25 When there is a COVID-19 vaccine widely available, would you approve or disapprove of employers requiring all employees to get the COVID-19 vaccine in order to come to work, unless they have a clear medical reason not to get vaccinated?
157

Strongly approve	1	279
Somewhat approve	2	
Somewhat disapprove	3	
Strongly disapprove	4	

Q26a AND Q26b ARE ROTATED BY FORM.

(ASK EVERYONE.)
Q26a How likely are you to get vaccinated for COVID-19 if your <u>out-of-pocket cost will be $20</u>?
929/932

I definitely will get vaccinated.	1	280
I probably will get vaccinated.	2	335
I probably will NOT get vaccinated.	3	
I definitely will NOT get vaccinated.	4	
I am completely undecided about whether I will get vaccinated.	5	

Q26b How likely are you to get vaccinated for COVID-19 if your <u>out-of-pocket cost will be $100</u>?
930/931

I definitely will get vaccinated.	1	308
I probably will get vaccinated.	2	
I probably will NOT get vaccinated.	3	336
I definitely will NOT get vaccinated.	4	
I am completely undecided about whether I will get vaccinated.	5	

Q26c How likely are you to get vaccinated for COVID-19 if the vaccine is <u>free and at no cost to you</u>?
933

I definitely will get vaccinated.	1	309
I probably will get vaccinated.	2	
I probably will NOT get vaccinated.	3	
I definitely will NOT get vaccinated.	4	
I am completely undecided about whether I will get vaccinated.	5	

Q26d The COVID-19 vaccine may require two doses to be effective. If you would need to get two doses of the COVID-19 vaccine, what impact would this have on you getting vaccinated?
934

Would make me more likely to get vaccinated	1	310
Would make me less likely to get vaccinated	2	
Would have no impact either way	3	

Q26e AND Q26f ARE ROTATED.

Q26e If the COVID-19 vaccine requires two doses and you had mild side effects from the first dose, what impact would this have on you getting the second dose?
935

Would make me much less likely to get the second dose	1	311
Would make me somewhat less likely to get the second dose	2	
Would not affect me getting a second dose	3	
Does not apply -- I would not get the vaccine at all	4	

Q26f If the COVID-19 vaccine requires two doses and it was a hassle or inconvenience for you to get the second dose, what impact would this have on you getting the second dose?
936

Would make me much less likely to get the second dose	1	312
Would make me somewhat less likely to get the second dose	2	
Would not affect me getting a second dose	3	
Does not apply -- I would not get the vaccine at all	4	

© 2021 Persad G et al. *JAMA Network Open.*

Q27a AND Q27b ARE ROTATED BY FORM.

Q27a Suppose a COVID-19 vaccine is proven to be effective at preventing COVID-19 in **60%** of people who receive the vaccine. What impact would this have on you getting vaccinated?

937/940

Would make me more likely to get vaccinated..1 313
Would make me less likely to get vaccinated 2 337
Would have no impact either way..3

Q27b Suppose a COVID-19 vaccine is proven to be effective at preventing COVID-19 in **75%** of people who receive the vaccine. What impact would this have on you getting vaccinated?

938/939

Would make me more likely to get vaccinated..1 314
Would make me less likely to get vaccinated 2
Would have no impact either way..3 338

(FORM A)

Q28a How important do you think it is that (**ROTATE**:) Democratic and Republican governors across the country work together to develop a coordinated response to COVID-19?

942

Very important 1 315
Fairly important 2
Only somewhat important 3
Not that important 4
Not important at all 5

(FORM B)

Q28b If (**ROTATE**:) Democratic and Republican governors across the country work together to develop a coordinated response to COVID-19, how much difference do you think it will make in controlling the spread of COVID-19 ?

944

Very big difference 1 316
Fairly big difference 2
Only somewhat of a difference 3
Not that much of a difference 4
No difference at all...5

(SHOW TO EVERYONE ON A SEPARATE SCREEN.)

These last few questions are for statistical purposes only.

QF1 The coronavirus situation has affected people in different ways. Please indicate whether any of the following apply to you. **(RANDOMIZE ITEMS. SHOW ON ONE SCREEN.)**

1. 945Someone in your household has a condition that makes them high-risk for COVID-19 317
2. 946You worry about a family member in a nursing home or long-term care facility 318
3. 947You or someone in your immediate family has gotten sick with the coronavirus (13024-Q28) 319

Apples to me...1
Does not apply to me...2

© 2021 Persad G et al. *JAMA Network Open.*

QF2 Which of the following best describes your main source of health insurance? **(DO NOT ROTATE.)**
158

I do not currently have health insurance coverage. 1 320
....
I have private health insurance through an employer. 2
I have private health insurance I buy on my own through Obamacare/the Affordable Care Act. 3
I have coverage through Medicaid or a similar government program for lower-income people. 4
I am on Medicare. 5
I have coverage through some other source. 6

QF3 Which of the following describes you?
159

Unmarried, not living with a partner 1 321
Unmarried, living with a partner 2
Married 3
Separated 4
Widowed 5
Divorced 6

QF4 Are you currently registered to vote?
948

Yes, registered to vote..1 322
....
No, not registered to vote 2
Not sure about registration status..3

QF5a Did you vote in the 2016 election for president? 323
949

Yes, voted ....1 **CONTINUE**
No, did not vote ....2 **SKIP TO QF6**

(ASK ONLY OF RESPONDENTS WHO SAY "YES, VOTED" IN QF5a.)

QF5b Which candidate did you vote for in the 2016 election for president? **(ROTATE PUNCHES 1 AND 2 ONLY.)**
950

Hillary Clinton (Democrat) 1 324
....
Donald Trump (Republican)..2
Gary Johnson (Libertarian)...3 **ANCHOR**
....
Jill Stein (Green Party) ....4 **ANCHOR**
Other ....5 **ANCHOR**

(ASK EVERYONE.)

QF6 Thinking about your general approach to issues, do you consider yourself to be…? **(ALTERNATE ORDER OF PUNCHES 1-5, 5-1.)**
951

Very conservative 1 325
Somewhat conservative 2
Moderate 3
Somewhat liberal 4
Very liberal 5
Not sure ....6 **ANCHOR**

QF7 Which cable news network do you watch most often, or do you not really watch cable news? **(RANDOMIZE PUNCHES 1-4.)**
160

CNN 1 326
Fox News Network 2
MSNBC 3
International news channels such as BBC, Al Jazeera, and Euronews..4

© 2021 Persad G et al. *JAMA Network Open.*

Do not watch cable news..5 **ANCHOR**

QF8 How often do you attend services at a church, synagogue, mosque, or other place of worship?
161

Never 1 327
Once a year 2
A few times a year 3
Once a month 4
About twice a month 5
Once a week or more often 6

QF9 And finally, may we please have your ZIP Code? **(REQUIRE 5-DIGIT RESPONSE.)** **328-332**

....

(SHOW ON A SEPARATE SCREEN.)
Thank you for your participation. That concludes this survey.

QF10 If there is anything else you would like to say or want us to know on the topics covered in the survey, please feel free to leave additional comments here. Also, please let us know if there were any questions in the survey that were unclear or confusing. **(DO <u>NOT</u> REQUIRE RESPONSE.)**

© 2021 Persad G et al. *JAMA Network Open.*

eTable 1. Gallup Survey vs COVID Collaborative Survey

	Gallup COVID-19 Panel	COVID Collaborative	
	Top-four priority group	Top-four priority group	Willingness to wait behind
**Vulnerable populations**			
Nursing home residents and staff	N/A	55.0%	96.2%
Republican<br>Democratic	N/A	57.8%**<br>51.2%**	96.1%<br>96.3%
Black<br>Non-Black	N/A	48.3%*<br>56.0%*	94.1%<br>96.5%
Minority<br>Non-minority	N/A	46.7%**<br>59.8%**	94.4%**<br>97.2%**
Bachelor's<br>Non-bachelors	N/A	56.2%<br>54.4%	97.7%*<br>95.4%*
Female<br>Male	N/A	59.0%**<br>50.6%**	96.4%<br>95.9%
Age ≥65<br>Age≤64	N/A	61.5%**<br>53.2%**	98.8%**<br>95.4%**

© 2021 Persad G et al. *JAMA Network Open.*

Medically vulnerable (co-morbidities)	78.6%	72.9%	95.6%
Republican<br>Democratic	82.4%<br>76.7%	73.9%<br>71.5%	95.3%<br>95.9%
Black<br>Non-Black	80.3%<br>78.5%	67.0%*<br>73.7%*	93.6%<br>95.9%
Minority<br>Non-minority	76.5%<br>79.5%	68.6%**<br>73.7%**	94.5%<br>96.2%
Bachelors Degree<br>Non-Bachelors	78.4%<br>78.7%	74.5%<br>72.0%	97.6%**<br>94.5%**
Female<br>Male	79.3%<br>77.9%	74.1%<br>71.6%	96.1%<br>95.2%
Age ≥65<br>Age≤64	74.3%<br>79.9%	76.4%<br>71.9%	96.7%<br>95.5%
Minority and other communities with higher COVID-19 burden	N/A	39.5%	84.9%
Republican<br>Democratic	N/A	33.8%*<br>47.1%*	82.1%**<br>88.8%**
Black<br>Non-Black	N/A	56.4%**<br>37.0%**	N/A

© 2021 Persad G et al. *JAMA Network Open.*

Minority<br>Non-Minority		46.3%**<br>35.5%**	N/A
Bachelors Degree<br>Non-Bachelors	N/A	39.1%<br>39.7%	85.6%<br>84.5%
Female<br>Male	N/A	40.1%<br>38.9%	87.6%**<br>82.0%**
Age ≥65<br>Age≤64	N/A	43.3%<br>38.3%	84.9%<br>84.9%
Adults in group settings	45.7%	N/A	N/A
Republican<br>Democratic	56.6%**<br>42.6%**	N/A	N/A
Black<br>Non-Black	44.7%<br>45.9%	N/A	N/A
Bachelors Degree<br>Non-Bachelors	45.2%<br>46.0%	N/A	N/A
Female<br>Male	51.0%*<br>40.1%*	N/A	N/A
Age >64<br>Age <65	50.6%<br>44.3%	N/A	N/A
Employment-based groups			

© 2021 Persad G et al. *JAMA Network Open.*

Health care workers	93.6%	80.0%	96.6%
Republican<br>Democratic	93.8%<br>95.7%	78.2%*<br>82.4%*	96.7%<br>96.6%
Black<br>Non-Black	90.3%<br>94.0%	72.1%**<br>81.2%**	95.1%<br>96.9%
Minority<br>Non-Minority	93.5%<br>93.6%	75.9%**<br>82.4%**	94.8%**<br>97.7%**
Bachelors Degree<br>Non-Bachelors	96.7%<br>91.9%	79.4%<br>80.3%	97.9%*<br>96.0%*
Female<br>Male	94.6%<br>92.5%	83.5%**<br>76.4%**	96.9%<br>96.3%
Age ≥65<br>Age≤64	94.0%<br>93.4%	94.0%<br>93.4%	99.1%**<br>96.0%**
Teachers and staff/ childcare workers	48.3%	32.2%	92.5%
Republican<br>Democratic	35.7%**<br>56.0%**	31.7%<br>32.8%	91.7%<br>93.7%
Black<br>Non-Black	53.8%<br>47.5%	30.2%<br>32.5%	89.4%<br>93.0%
Bachelors Degree<br>Non-Bachelors	50.8%<br>46.9%	33.4%<br>31.6%	93.3%<br>92.2%
Minority	50.1%	30.0%	91.2%

© 2021 Persad G et al. *JAMA Network Open.*

Non-Minority	47.5%	33.4%	93.3%
Female<br>Male	46.7%<br>50.0%	33.0%<br>31.3%	94.2%**<br>90.8%**
Age ≥65<br>Age≤64	51.5%<br>47.3%		93.7%<br>92.2%
Non-health essential workers	47.1%	N/A	N/A
Republican<br>Democratic	36.3%**<br>53.1%**	N/A	N/A
Black<br>Non-Black	47.1%<br>47.0%	N/A	N/A
Bachelors Degree<br>Non-Bachelors	50.9%<br>45.0%	N/A	N/A
Female<br>Male	52.2%**<br>41.8%**	N/A	N/A
Age >64<br>Age <65	52.1%<br>45.6%	N/A	N/A
Grocery store workers	N/A	14.4%	85.9%
Republican<br>Democratic	N/A	13.1%<br>16.1%	85.5%<br>86.5%

© 2021 Persad G et al. *JAMA Network Open.*

Black<br>Non-Black	N/A	14.7%<br>14.3%	83.8%<br>86.3%
Minority<br>Non-Minority	N/A	15.4%<br>13.8%	86.7%<br>85.5%
Bachelors Degree<br>Non-Bachelors	N/A	13.4%<br>14.9%	87.7%<br>85.0%
Female<br>Male	N/A	13.9%<br>14.9%	86.5%<br>85.3%
Age ≥65<br>Age≤64	N/A	13.7%<br>14.7%	83.3%<br>86.7%
Restaurant, bar, and gym workers	N/A	8.9%	74.2%
Republican<br>Democratic	N/A	8.3%<br>9.9%	76.0%<br>71.8%
Black<br>Non-Black	N/A	10.1%<br>8.8%	73.3%<br>74.3%
Minority<br>Non-Minority	N/A	11.5%**<br>7.5%**	76.2%<br>73.0%
Bachelors Degree<br>Non-Bachelors	N/A	7.8%<br>9.5%	74.9%<br>73.8%
Female<br>Male	N/A	9.3%<br>8.4%	76.0%<br>72.3%

© 2021 Persad G et al. *JAMA Network Open.*

Age ≥65<br>Age≤64	N/A	6.7%*<br>9.9%*	69.7%*<br>75.4%*
People in prisons/prison guards	N/A	8.2%	56.4%
Republican<br>Democratic	N/A	7.7%<br>8.8%	55.9%<br>56.9%
Black<br>Non-Black	N/A	10.6%<br>7.8%	58.3%<br>56.1%
Minority<br>Non-Minority	N/A	9.0%<br>7.7%	58.9%*<br>54.9%*
Bachelors Degree<br>Non-Bachelors	N/A	8.2%<br>8.1%	59.8%<br>54.7%
Female<br>Male	N/A	9.1%<br>7.1%	58.5%<br>54.0%
Age ≥65<br>Age≤64	N/A	6.3%<br>9.0%	47.5%**<br>58.7%**
Participants in COVID-19 research	32.8%	26.3%	89.7%
Republican<br>Democratic	32.2%<br>33.9%	27.2%<br>25.0%	88.5%<br>91.3%
Black	30.4%	27.4%	86.7%

© 2021 Persad G et al. *JAMA Network Open.*

Non-Black	33.2%	26.1%	90.2%
Minority<br><br>Non-Minority	29.5%<br><br>34.1%	27.3%<br><br>25.7%	89.3%<br><br>89.9%
Bachelors Degree<br><br>Non-Bachelors	30.1%<br><br>34.3%	26.7%<br><br>26.0%	91.1%<br><br>89.0%
Female<br><br>Male	33.1%<br><br>32.6%	24.4%<br><br>28.3%	90.5%<br><br>88.9%
Age ≥65<br><br>Age≤64	31.6%<br><br>33.2%	30.0%*<br><br>24.7%*	91.6%<br><br>89.2%
Age groups			
Healthy adults 65 and over	35.6%	27.6%	87.6%
Republican<br><br>Democratic	44.8%**<br><br>24.7%**	30.0%<br><br>24.4%	88.2%<br><br>86.7%
Black<br><br>Non-Black	39.9%<br><br>35.1%	22.5%<br><br>28.4%	85.6%<br><br>87.8%
Minority<br><br>Non-Minority	36.2%<br><br>35.5%	25.8%<br><br>28.7%	86.7%<br><br>88.0%
Bachelors Degree<br><br>Non-Bachelors	35.5%<br><br>35.7%	28.1%<br><br>27.4%	88.9%<br><br>86.9%
Female	25.9%**	24.8%**	87.8%

© 2021 Persad G et al. *JAMA Network Open.*

Male	45.8%**	30.6%**	87.3%
Age ≥65<br>Age≤64	23.7%**<br>39.1%**	23.3%*<br>28.8%*	N/A
Healthy adults age 30-65	N/A	8.6%	60.8%
Republican<br>Democratic	N/A	9.6%<br>7.2%	62.9%<br>58.2%
Black<br>Non-Black	N/A	10.0%<br>8.4%	64.0%<br>60.3%
Minority<br>Non-Minority	N/A	10.6%*<br>7.4%*	70.0%**<br>57.0%**
Bachelors Degree<br>Non-Bachelors	N/A	8.9%<br>8.5%	57.8%<br>62.2%
Female<br>Male	N/A	6.8%**<br>10.5%**	61.2%<br>60.4%
Age ≥65<br>Age≤64	N/a	2.0%**<br>10.4%**	44.2%**<br>66.5%**
Young adults age 19-29	N/A	5.5%	53.1%
Republican<br>Democratic	N/A	5.8%<br>5.1%	54.0%<br>51.9%
Black	N/A	6.7%	62.0%**

© 2021 Persad G et al. *JAMA Network Open.*

Non-Black		5.4%	51.8%**
Minority<br>Non-Minority	N/A	5.8%<br>5.3%	61.6%**<br>48.4%**
Bachelors Degree<br>Non-Bachelors	N/A	6.2%<br>5.2%	49.7%*<br>54.9%*
Female<br>Male	N/A	3.1%**<br>8.1%**	55.3%<br>50.7%
Age ≥65<br>Age≤64	N/A	2.2%**<br>5.4%**	40.5%**<br>56.8%**
Healthy adults age 18-65	5.1%	N/A	N/A
Republican<br>Democratic	7.4%<br>3.4%	N/A	N/A
Black<br>Non-Black	5.4%<br>5.1%	N/A	N/A
Bachelors Degree<br>Non-Bachelors	3.2%<br>6.1%	N/A	N/A
Female<br>Male	4.3%<br>6.0%	N/A	N/A
Age ≥60<br>Age≤59	5.9%<br>4.9%	N/A	N/A
Children age 0-18	13.1%	N/A	N/A

© 2021 Persad G et al. *JAMA Network Open.*

Republican<br>Democratic	9.2%<br>15.7%	N/A	N/A
Black<br>Non-Black	8.0%<br>13.6%	N/A	N/A
Minority<br>Non-Minority	13.9%<br>12.7%	N/A	N/A
Bachelors Degree<br>Non-Bachelors	9.1%*<br>15.2%*	N/A	N/A
Female<br>Male	12.9%<br>13.4%	N/A	N/A
Age ≥60<br>Age≤59	16.3%<br>12.2%	N/A	N/A
Teenagers (13-18)	N/A	6.7%	60.0%
Republican<br>Democratic	N/A	7.1%<br>6.2%	59.5%<br>60.8%
Black<br>Non-Black	N/A	10.2%<br>6.2%	64.9%<br>59.3%
Minority<br>Non-Minority	N/A	9.6%**<br>5.0%**	66.6%**<br>56.2%**
Bachelors Degree<br>Non-Bachelors	N/A	5.8%<br>7.2%	55.5%**<br>62.3%**
Female	N/A	5.3%*	62.9%*

© 2021 Persad G et al. *JAMA Network Open.*

Male		8.3%*	56.9%*
Age ≥65<br>Age≤64	N/A	3.2%**<br>7.7%**	44.0%**<br>64.4%**
Young children (12 and under)	N/A	14.2%	69.5%
Republican<br>Democratic	N/A	15.8%*<br>12.1%*	69.4%<br>69.7%
Black<br>Non-Black	N/A	13.8%<br>14.3%	75.4%*<br>68.6%*
Minority<br>Non-minority	N/A	17.5%**<br>12.3%**	76.4%**<br>65.5%**
Bachelors Degree<br>Non-Bachelors	N/A	12.1%<br>15.3%	65.1%**<br>71.7%**
Female<br>Male	N/A	13.6%<br>14.9%	70.7%<br>68.2%
Age ≥65<br>Age≤64	N/A	5.8%**<br>16.5%**	52.9%**<br>73.9%**

*Statistically significant at p < .05
**Statistically significant at p<.01

© 2021 Persad G et al. *JAMA Network Open.*

eTable 2. Support for Prioritizing Communities Hard Hit by COVID-19

	Gallup COVID-19 Panel	COVID Collaborative (Hart)
Overall	74.2%	84.9%
Republican	60.4%**	82.1%**
Democrat	87.7%**	88.8%**
Very Conservative	N/A	76.9%**
Not Very Conservative	N/A	86.4%**

*Statistically significant at p < .05
**Statistically significant at p < .01

© 2021 Persad G et al. *JAMA Network Open.*

eTable 3. Reasons for Prioritization (Select 2)

Focus on what will most prevent the spread of the virus	78.4%
Very Conservative<br>Not very conservative	73.3%*<br>79.3%*
Minority<br>Non-minority	78.9%<br>78.0%
Focus on what will prevent the most deaths	72.1%
Very Conservative<br>Not very conservative	71.1%<br>72.3%
Minority<br>Non-minority	65.8%**<br>75.8%**
Focus on what will protect the most people from long-term health complications	68.9%
Very Conservative<br>Not very conservative	68.6%<br>68.9%
Minority<br>Non-minority	65.0%*<br>71.2%*
Focus on protecting frontline workers	63.8%
Very Conservative<br>Not very conservative	65.9%<br>63.5%
Minority<br>Non-minority	62.1%<br>64.8%
Focus on what will prevent the most lost years of life	22.4%
Very Conservative<br>Not very conservative	23.5%<br>22.2%
Minority<br>Non-minority	26.5%*<br>20.0%*
Focus on what will most help the economic recovery	36.5%
Very Conservative<br>Not very conservative	52.7%**<br>33.3%**
Minority<br>Non-minority	37.9%<br>35.7%

*Statistically significant at $p < .05$
**Statistically significant at $p<.01$

© 2021 Persad G et al. *JAMA Network Open.*
